# Choroidal thickness in lamellar macular holes

**DOI:** 10.1007/s00417-020-04922-2

**Published:** 2020-09-18

**Authors:** Magdalena Kal, Mateusz Winiarczyk, Stanisław Głuszek, Jerzy Mackiewicz

**Affiliations:** 1 Institute of Medical Sciences of Jan Kochanowski University in Kielce, Kielce, Poland; 2 Ophthalmic Clinic of the Provincial Hospital in Kielce, Kielce, Poland; 3 Department of Vitreoretinal Surgery, Medical University of Lublin, Lublin, Poland; 4 Collegium Medicum of Jan Kochanowski University in Kielce, Kielce, Poland; 5 Oncological, Endocrinological and General Surgery Clinic of the Provincial Hospital in Kielce, Kielce, Poland

**Keywords:** Lamellar macular hole, Choroid, Choroidal thickness, Swept-source OCT, Optical coherence tomography

## Abstract

**Purpose:**

(1) To assess the thickness of the central choroid (BM-CSI) in swept-source optical coherence tomography (SS-OCT) examination of lamellar macular holes (LMHs). (2) To establish correlations between the thickness of the central choroid (BM-CSI) in the LHM and the parameters of best-corrected visual acuity and reading vision in patients with LMH.

**Methods:**

This prospective case-control study assessed a group of 30 patients (30 eyes) with LMHs and a control group of 45 patients (90 eyes). The thickness of the central choroid (BM-CSI) was measured with an SS-OCT device. The average choroidal thickness in the fovea was defined as average thickness in the central area of 1000 μm in diameter, according to the Early Treatment Diabetic Retinopathy Study (ETDRS). The results were correlated with best-corrected visual acuity (BCVA), and reading vision.

**Results:**

The average choroidal thickness in the study group (SG) with LMH was 160.34 μm (SD = 77.1), whereas in the control group (CG), it was 225.11 μm (SD = 93.8). The difference of 64.77 μm was statistically significant (*p* < 0.05). The BCVA was within the range between 0.7 (logMAR) and 0.1 (logMAR), with an average of 0.36 (logMAR) (SD = 0.23). Reading vision was within the range between − 0.2 (logMAR) and 0.3 (logMAR), with an average of 0.27 (logMAR) (SD = 0.12). A significant correlation between BCVA and the choroid (BM-CSI) was found. The correlation coefficient is average (*r* = 0.44) and positive. With better BCVA, a significantly thicker choroid (BM-CSI) can be observed. No significant correlation between BM-CSI and reading vision was found. The correlation coefficient value is minor (*r* = − 0.289), whereas lower values of BM-CSI can be observed with worse reading vision.

**Conclusion:**

We suggest that the choroid may take part in the pathogenesis of LMH development. Its significant thinning may be responsible for the ischemic degenerative mechanism degenerating outer layers of retina, apart from tractional mechanism.

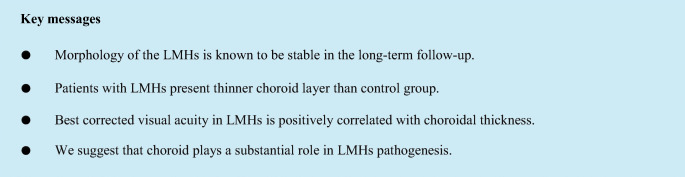

## Introduction

The choroid of the eye plays a key role in its physiology by distributing nutrients into the retinal pigment epithelium (RPE) and its outer layers and by regulating the eye growth [1].

The assessment of the choroid is very important in macular diseases, and it is now possible via swept-source optical coherence tomography (SS-OCT) [2–4]. We can observe an increase in choroid thickness in certain eye diseases, which include central serous chorioretinopathy, Vogt-Koyanagi-Harada disease, multiple evanescent white dot syndrome, adult-onset vitelliform macular dystrophy, and parafoveal telangiectasia type 2. Thinning of the choroid is found in the presence of high myopia, in diabetes, subretinal pseudodrusen, or subretinal drusenoid deposits [2].

The latest technology—SS-OCT with a tuned light source—allows the examination of even the deeper layers of the eye such as the choroid [5]. Penetration of light waves reaches up to the sclera. The greatest advantage of this method is the fastest scanning speed available—370,000 scans A/s [6]. Images of the choroid obtained in the latest SS-OCT devices match the histological structure of the tissue. Thanks to the properties described above, SS-OCT allows simultaneous acquisition of a high-quality image of the vitreous humor, retina, choroid, and sclera.

In our examination, we assessed the thickness of the central choroid (BM-CSI) in lamellar macular holes (LMHs). Differentiation of non-full-thickness macular holes (NFTMHs) is possible via OCT, while in biomicroscope testing, the appropriate diagnosis regarding these pathologies is only possible in only 28 to 37% of cases [7, 8].

**Fig. 1:**
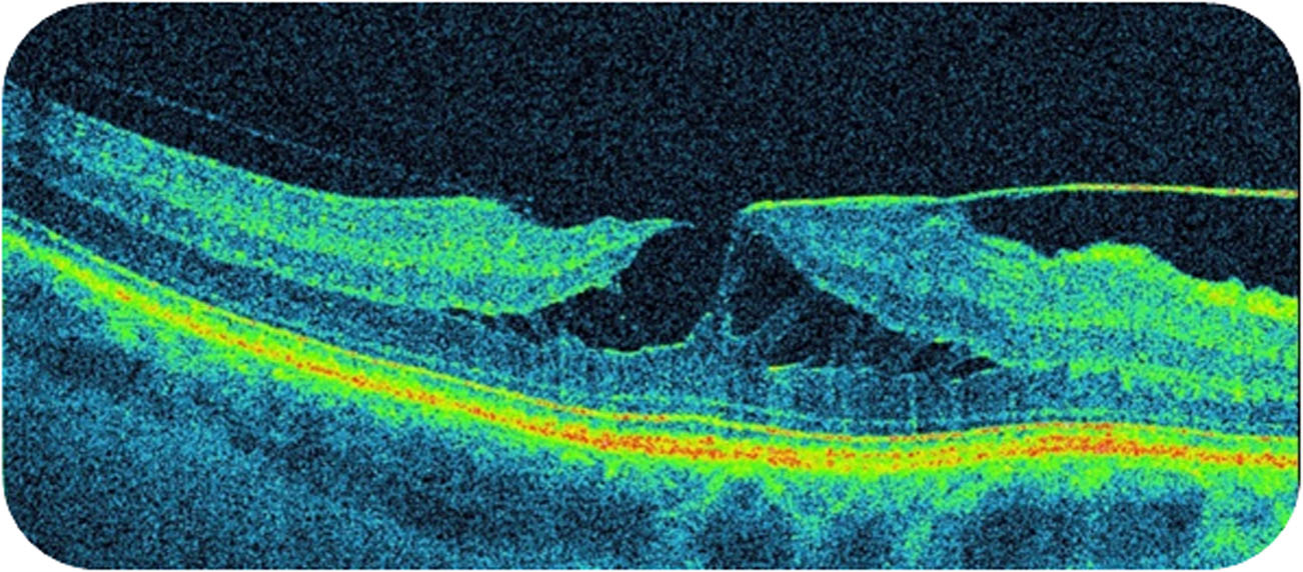
Lamellar macular hole with epiretinal membrane in SS-OCT examination

An LMH is characterized by the irregular contour of the fovea, located below the outer plexiform layer and the separation of inner and outer layers within the central area of the retina. Additionally, cyst-like areas are present around the fovea. There is no full-thickness macular hole (FTMH). The thickness of the central retina may be normal, decreased, or increased. The epiretinal membrane (ERM) is found in 100% LMH, whereby the photoreceptor layer may be damaged. Visual acuity in LMH may be correct or lowered [9–11]. Identification of LMH is made on the basis of medical signs and OCT examination (Fig. 1).

Undoubtedly the greatest role in the development NFTMHs is played by posterior vitreous detachment (PVD). This involves both LMHs and macular pseudoholes. Here, fragments of the internal limiting membrane (ILM) become separated, which stimulates Müller cell proliferation and the creation of macular edema. By shrinking, the edema causes anterior-posterior vitreomacular traction, tangential to the surface of the retina [12–17]. The degenerative mechanism of LMH development also cannot be excluded. It can be present in cases of LMH with the presence of epiretinal proliferation (LHEP) [18, 19]. LMH may be of idiopathic character or develop as a result of cataract surgery, nearsightedness, uveitis, age-related macular degeneration (AMD), and retinal detachment.

## Materials and methods

This prospective case-control study assessed a study group (SG) of 30 patients (30 eyes) with LMHs. The inclusion criterion for the SG was the LMH presence, confirmed by fundus indirect ophthalmoscopy and OCT examination. We performed a prospective study which included patients with the LMH, with a minimum follow-up of 36 months. The definition of LMH was as previously described [8–11, 20–22]. In the course of the study, all LMHs were assessed for the stability or signs of the progression of the lesion. The stability of the LMH was defined as a lack of progression in the lesion diameter in the widest and narrowest point, no progression to full-thickness MH, and the same proportions in the en face retinal thickness map in the OCT. At the follow-up visit, the choroidal thickness measurement was carried out in all SG patients.

Automatic maps of thickness and volume of retinal layers, and ETDRS choroidal thickness maps comprising nine sectors and built in the SS-OCT device, served for automatic assessment of the choroidal thickness (BM-CSI). The diameter of the internal ring was 3 mm, and the outer ring was 6 mm. The average thickness of the choroid in the fovea is defined as the average choroidal thickness in the central area of 1000 μm in diameter, according to ETDRS. Automatic measurement contains 96 images for each B-scan, which are obtained in 1 s.

Control group consisted of 45 patients (90 eyes), age matched with the SG, that shown no ocular pathologies nor previous ocular surgery, apart from the cataract extraction. These were patients scheduled for a routine cataract surgery.

The following diseases constituted exclusion criteria from the SG: diabetic retinopathy, state after a central retinal vein occlusion, past eye injuries, past retinal surgery, high myopia (> 6D), past intraocular inflammation, age-related macular degeneration, glaucoma, and insufficient number of check-ups.

Full ophthalmological examinations were conducted in all patients, including thorough ophthalmological history, family history and general illnesses, best-corrected visual acuity and reading vision examination (logMAR scale), eye fundus examination via slit lamp with a 78 D lens (Volk), and assessment of the fundus via OCT (SS-OCT Triton, Topcon) that allow the assessment of BM-CSI—automatic measurement of the choroidal thickness. Patients were examined in the afternoon between 3 and 5 pm by a single investigator (MW).

### Statistical analysis

In relation to quantitative properties, the normality of the distribution was assessed through the Shapiro-Wilk test. To compare the average values of BM-CSI in both compared groups, patients with holes and the CG, Student’s *t* test was used. To determine the correlation between logMAR for the best-corrected visual acuity (BCVA) and reading vision with BM-CSI values, R Spearman correlations were used. The results regarded as statistically significant were those for which the *p* values were at the level of *p* ≤ 0.05. Statistical calculations were conducted via STATISTICA 13.3. PL (StatSoft) package.

## Results

### Demographic data

A group of 30 patients (30 eyes) (SG) was assessed with LMH, 26 of which were women (86.67%) and four were men (13.33%), with an average age of 70.43 (SD = 9.93). The control group (CG) comprised 45 healthy persons (90 eyes), including 28 women (62.23%) and 17 men (37.77%), with an average age of 66.26 years (SD = 11.25).

LMH were followed up for 36 months, showing no significant signs of progression in OCT examination as described in “Materials and methods” section. At the last visit, choroidal thickness was measured. The refraction of all examined eyes was between − 5.0 Dsph and + 5.0 Dsph, with a mean refractive error of − 0.6Dsph (SD = 2,40). None of the patients presented atrophic retinal changes. Four patients underwent cataract operation with intraocular lens implantation (13.34%), and 26 (86.66%) patients were phakic. BCVA was within the range of 0.7 (logMAR) and 0.1 (logMAR). The average BCVA was 0.36 (logMAR) (SD = 0.23).

Reading vision was within the range of − 0.2 (logMAR) and 0.3 (logMAR), with an average of 0.27 (SD = 0.12) (logMAR).

No statistically significant gender differences were observed for the average age of patients in each group, the SG and the CG (*p* = 0.105).

### Choroidal thickness relation to BCVA and reading vision

**Fig. 2:**
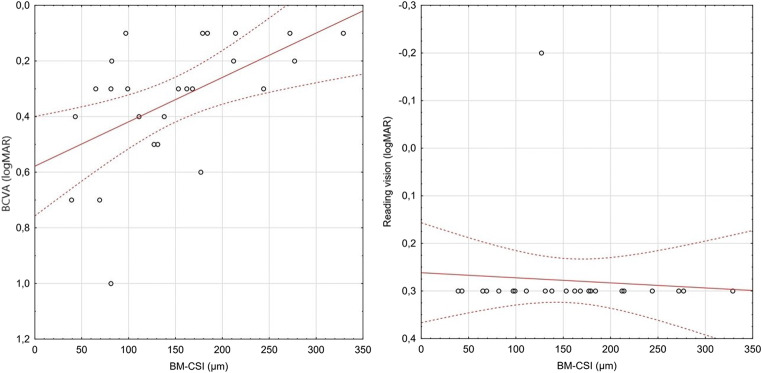
Correlations between BM-CSI and BCVA/reading vision in patients with LMH

A statistically significant correlation between BCVA and the choroid (BM-CSI) was found (*p* = 0.0179). The correlation coefficient is average (*r* = 0.44) and positive. With better BCVA, a significantly thicker choroid (BM-CSI) can be observed (Fig. 2).

No significant correlation between BM-CSI and reading vision was found. The correlation coefficient value is minor (*r* = − 0.289), whereas lower values of BM-CSI could be observed with worse reading vision (*p* = 0.153) (Fig. 2).

**Fig. 3:**
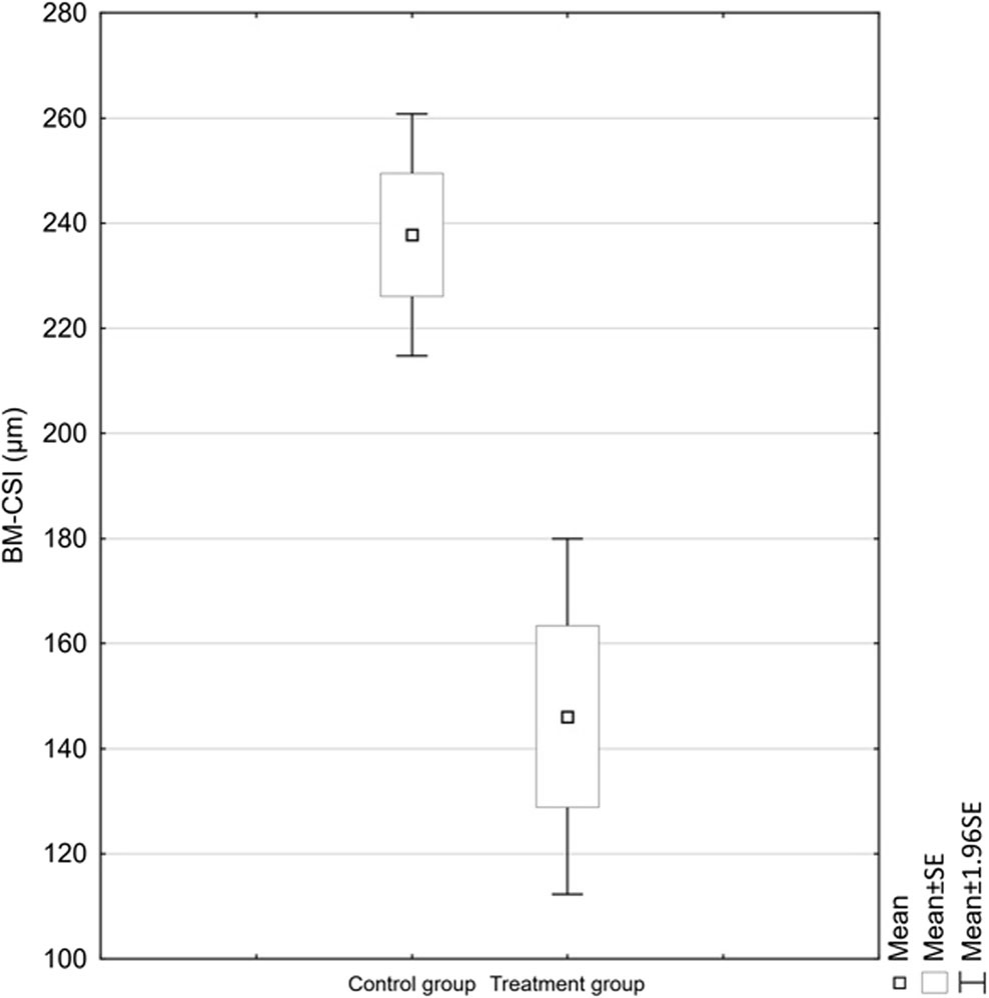
Average BM-CSI values in the compared groups

The average choroidal thickness in the study group (SG) with LMH was 160.34 μm (SD = 77.1), whereas in the CG, it was 225.11 μm (SD = 93.8). The difference of 64.77 μm was statistically significant (*p* = 0.002) (Fig. 3).

## Discussion

Over the last few years, the imaging techniques of the central retina and the deeper choroid via a swept-source OCT (SS-OCT) have allowed better visualization and understanding of the vitreomacular interface disorders [7]. It enables detection and monitoring of non-full-thickness macular holes, among which we distinguish LMHs and macular pseudoholes (MPHs) [20–22].

In our study, demographic distribution, workflow, criteria of morphological assessment in the assessment of natural LMH development, as well as the equipment used were similar to procedures used in other publications [8, 11, 22–27].

The choroid comprises three layers: the outer large choroidal vessel layer – Haller’s layer, inner medium-sized vessel layer – Sattler’s layer, and small vessels – choriocapillaris. Taking into account the unit of mass, the choroid possesses the highest blood flow rate value of all anatomic structures. It is the only metabolic source of the eye within the avascular zone of the fovea. It is responsible for the blood supply into the retinal pigment epithelium (RPE), and outer layers of the retina and part of the optic nerve can also be supplied by the choroid [4]. The choroid takes part in the nourishing and oxygenation of the retina, as well as in the regulation of eyeball growth. A local increase in its thickness may suggest its higher metabolism [28].

In Parravano’s observations, the causes of disorders in the outer LMH layers are vitreomacular adhesion (VMA) and posterior vitreous detachment (PVD) [29]. Thinning of the retina in LMH is considered as a result of foveal cyst separation in the course of the vitreous humor changes during PVD, with retinal outer layers not affected. LMH may result from the disrupted formation of a full-thickness macular hole [8, 30–32]. Another factor in the development of idiopathic LMH is the epiretinal membrane (ERM) [33, 34]. The presence of ERM worsens visual acuity likely due to swelling of the retina [29, 35, 36], reduction in parafoveal blood flow, and morphological changes in the outer retina [37]. Studies by some authors also suggest a degenerative mechanism of LMH development. It can be present in cases of LMH with the presence of epiretinal proliferation (LHEP) [18, 19].

In our study, we analyzed a homogenous group of nonprogressive LMH, with at least 36 months of documented stability in OCT.

There is scarce evidence in the existing literature assessing the choroidal thickness in LMH. With SS-OCT, which has a higher resolution than SD-OCT, it became possible to carry out choroid measurements using three-dimensional ETDRS choroidal thickness maps [28, 38]. ETDRS choroid thickness maps were proven to be repeatable [29]. Furthermore, automatic measurements of the choroid are more accurate than manual measurements, where significant differences between the examiners of the designated patient groups were reported up to 32 μm [39]. The analysis of the choroid in patients with LMH allows us to conclude that changes in this highly vascularized layer may affect the LMH growth. In our study, we report lower BM-CSI values in LMH patients than in healthy subjects. In the studied group, the average subfoveal BM-CSI value was 160.3 μm, compared with 225.1 μm in the CG. The difference proved to be statistically significant (*p* = 0.002). This suggests choroid involvement in LMH creation. One may hypothesize that impaired choroidal blood flow induces ischemic changes in the retina, leading to the formation of cysts and retinal layers splitting.

In previous studies, various choroidal thickness results for the subfoveal area in healthy subjects were obtained with SS-OCT ranging from 192 [40] to 448 μm [38] in different age groups. Choroidal thickness is reported to decrease by 14 to 15.6 μm per each decade of life [28, 40–44]. The literature reports choroidal thickness fluctuations depending on the time of day, with the greatest thickness at 3 am, and the minimal thickness at 6 pm [45]. Patients in our group were examined in the afternoon, between 3 and 5 pm. According to our state of knowledge, similar examinations in patients with LMH were not carried out; therefore, it is not possible to compare our results to other studies.

Automatic measurement of choroidal thickness was performed in patients after uncomplicated cataract surgery [46], after anti-VEGF injections, in pachychoroid neovasculopathy (PNV) [47], in central serous chorioretinopathy [48], in dry AMD [2], and in parafoveal telangiectasia type 2 [49]. Many authors attempt to understand the significance of the choroid in AMD development. Lu et al. determined that the thinning of the choroid in patients with dry AMD due to the thinning of choriocapillaris and the Sattler’s medium-sized vessel layer leads to RPE atrophy. Due to the age of patients with AMD, it is also worth taking into account the natural age-related decrease in blood flow in the choroid, which takes place in the aforementioned vascular layers of the choroid. AMD development is undoubtedly affected by the decrease in the capability of supplying oxygen by the choroid to the PRE and outer layers of retina, as well as the build-up of lipofuscin in the RPE/Bruch’s layer [2, 50].

The thickness of the choroid in high myopia was also assessed, where thinning can lead to the photoreceptor cell death and vision loss as a consequence. In the case of myopic eyes, the excessive axial eye elongation may lead to biomechanical stretching and thinning of the choroid, retina, and sclera. Different results of choroid measurement were obtained in diseases described as “pachychoroid.” These include central serous chorioretinopathy (CSC), polypoidal choroidal vasculopathy (PCV) and Vogt-Koyanagi-Harada disease (VKH) [51]. Increases in the thickness of the choroid in these pathologies stem from the expansion of the large vessel layer—Haller’s layer (pachyvessels)—which is accompanied by a thinning of the choriocapillaris and Sattler’s layer with or without disorders of RPE covering the pachyvessels. The thickness of the choroid is the key criterion in defining the phenotype of pachychoroid disease. There are also characteristic morphological changes here which imply structural and functional changes in the choroid as a key pathophysiological mechanism [52].

There are also studies showing that choroidal thickness can be affected in systemic diseases, such as anemia or carotid artery stenosis, showing its significant reduction [53, 54].

## Conclusion

To the best of our knowledge, we are the first group to report the correlation of choroidal thickness with LMH. In our study, we observed high stability of the LMHs; none of our SG patients shown any signs of the progression of the lesion. After the analysis of the aforementioned reports regarding the choroidal thickness and its possible contribution to ocular and systemic diseases, it is possible to express a hypothesis that the choroid can play a role in the pathogenesis of LMH. Significant choroidal thinning can be responsible for the ischemic degenerative mechanism of the outer layers of the retina in LMH, apart from the tractional mechanism. Further research is needed in patients with LMH to better understand the etiopathogenesis of this disease.

## Data Availability

Not applicable.
